# Outcomes Associated With Osteochondral Allograft Transplantation in Dogs

**DOI:** 10.3389/fvets.2021.759610

**Published:** 2021-12-24

**Authors:** Samuel P. Franklin, Aaron M. Stoker, Sean M. Murphy, Michael P. Kowaleski, Mitchell Gillick, Stanley E. Kim, Michael Karlin, Alan Cross, James L. Cook

**Affiliations:** ^1^Colorado Canine Orthopedics and Rehab, Colorado Springs, CO, United States; ^2^Thompson Laboratory for Regenerative Orthopaedics, University of Missouri, Columbia, MO, United States; ^3^WestVet Animal Emergency and Specialty Center, Garden City, ID, United States; ^4^Department of Clinical Sciences, Cummings School of Veterinary Medicine, Tufts University, North Grafton, MA, United States; ^5^Toronto Veterinary Emergency and Referral Hospital, Toronto, ON, Canada; ^6^Department of Small Animal Clinical Sciences, University of Florida, Gainesville, FL, United States; ^7^BluePearl Pet Hospital, Atlanta, GA, United States

**Keywords:** osteochondral, allograft, transplants, cartilage repair, dogs, osteochondrosis, osteochondritis dissecans

## Abstract

The purpose of this study was to retrospectively characterize outcomes and complications associated with osteochondral allograft transplantation for treating chondral and osteochondral lesions in a group of client-owned dogs with naturally-occurring disease. Records were reviewed for information on signalment, treated joint, underlying pathology (e.g., osteochondritis dissecans; OCD), and type, size, and number of grafts used. Complications were classified as “trivial” if no treatment was provided, “non-surgical” if non-surgical treatment were needed, “minor surgical” if a minor surgical procedure such as pin removal were needed but the graft survived and function was acceptable, or “major” if the graft failed and revision surgery were needed. Outcomes were classified as unacceptable, acceptable, or full function. Thirty-five joints in 33 dogs were treated including nine stifles with lateral femoral condyle (LFC) OCD and 10 stifles with medial femoral condyle (MFC) OCD treated with osteochondral cylinders or “plugs.” There were 16 “complex” procedures of the shoulder, elbow, hip, stifle, and tarsus using custom-cut grafts. In total there were eight trivial complications, one non-surgical complication, two minor surgical complications, and five major complications for a total of 16/35 cases with complications. Accordingly, there were five cases with unacceptable outcomes, all of whom had major complications while the other 30 cases had successful outcomes. Of the 30 cases with successful outcomes, 15 had full function and 15 had acceptable function. Based on these subjective outcome assessments, it appears osteochondral allograft transplantation is a viable treatment option in dogs with focal or complex cartilage defects. However, no conclusions can be made regarding the inferiority or superiority of allograft transplantation in comparison to other treatment options based upon these data.

## Introduction

Cartilage damage that causes pain or lameness is common in people and dogs, either secondary to osteoarthritis in which case cartilage loss is typically diffuse, or as focal cartilage defects secondary to osteochondrosis, trauma, or athletic injury (1–7). Many treatment options for cartilage damage are used in both human and canine medicine, including non-surgical management, osteochondral autografting, or synthetic resurfacing with focal, unicompartmental, or total joint replacement systems (8–14). However, osteochondral allograft (OCA) transplantation is a treatment option used in people, which has not been described for use in clinical canine patients (15–18).

Osteochondral allograft transplantation involves the transfer of viable, also termed “fresh,” osteochondral tissue from a deceased tissue donor to a recipient patient to resurface articular defects with intact hyaline articular cartilage (19–21). The process starts with aseptic recovery of the donor tissue followed by preservation at a tissue bank. Donors, tissues, and media are assessed for infectious diseases prior to tissue “clearance” of infection. Once cleared, the tissue is matched and allocated to a recipient based on anatomic surface(s) to be treated and the relative size of the donor and recipient joints. Blood type and major histocompatibility complex are not criteria for OCA matching because chondrocytes in intact hyaline cartilage are immune-privileged and antigenic marrow elements are sufficiently removed during tissue storage and graft preparation procedures to avoid immune-mediated rejection (20). Marrow elements are effectively removed because they die during culture, are shed into the tissue culture media, and are removed with changes of media. In addition, the cancellous bone portion is lavaged prior to surgical implantation to remove any remaining marrow elements. Properly preserved and prepared OCAs elicit accommodation by the immune system, rather than rejection (22, 23). Accordingly, human patients receiving osteochondral allografts do not require immunosuppression.

Osteochondral allograft transplantation has been reported to be effective for resurfacing cartilage defects in the human knee, ankle, hip, shoulder, and elbow (17, 18, 24–26). Resurfacing is most commonly performed using cylindrical OCA cores, or “plugs,” using essentially the same equipment and techniques used for osteochondral autograft transfer (OAT). As surgical techniques have advanced, OCA transplantation has been used to treat larger portions of joints and more complex cases including total biologic joint resurfacing (17, 18, 27). Outcomes after OCA transplantation in people are significantly affected by a number of factors including underlying pathologic change, specific joint treated, chondrocyte viability at time of implantation, patient age, body mass index, tobacco use, number of grafts transplanted, and comorbidities (28–31). Functional success rates and 2- to 5-year survival for unipolar single-surface OCA plugs consistently exceed 90%. With implementation of novel graft preservation, cutting and preparation techniques, and comprehensive pre- and post-operative protocols, success rates and 2- to 5-year survival for large single-surface, multi-surface and bipolar (opposing articulating surfaces) shell grafts now exceed 80% (18, 19, 27, 32).

Given the success of OCA transplantation in humans, OCA transplantation may have applicability in dogs, particularly when considering that many recent advances in OCA transplantation have been developed and validated using pre-clinical canine models (15–17, 19, 33, 34). However, data from experimental studies in research dogs with induced cartilage defects are not necessarily representative of success rates that could be expected for dogs with naturally-occurring disease. To the authors' knowledge, OCA transplantation has not been described in clinical canine patients. As a result, veterinary surgeons considering use of OCA transplantation for canine patients cannot make clinical-evidence-based decisions regarding indications and techniques or provide data regarding complications, success rates, and prognoses to pet owners. Therefore, the purpose of this study was to assess outcomes associated with OCA transplantation in dogs for treatment of naturally-occurring cartilage defects.

## Materials and Methods

This retrospective study involved acquisition of data for all dogs undergoing OCA transplantation using OCAs provided by a single canine tissue bank (The Thompson Laboratory for Regenerative Orthopaedics, University of Missouri) and for whom follow-up data were available. All recipient dogs were client-owned and informed consent was obtained prior to surgery.

With institutional animal care and use committee approval, all tissues were recovered from donor dogs, and specifically from the homologous joints that were to be treated in the recipients, humanely euthanized for reasons unrelated to tissue recovery and use. Canine tissue donors were documented to have been vaccinated for relevant infectious agents and medical records were reviewed to rule out infectious or neoplastic disorders present at time of euthanasia. Osteochondral tissues were recovered using strict aseptic technique immediately following euthanasia (i.e., within 30 min of euthanasia), measured, and immediately placed in a proprietary medium and stored in accordance with a validated protocol (Missouri Osteochondral Preservation System; MOPS®) (15, 16, 31). Samples of tissue and media for each OCA underwent microbial testing in aerobic and anaerobic conditions. All cultures were maintained for 14 days.

**Figure 1:**
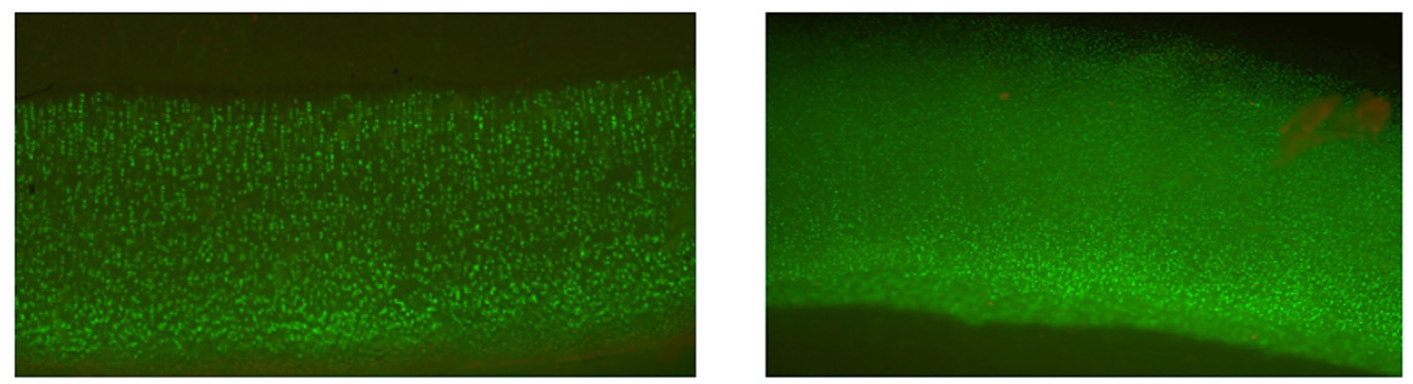
Cellular viability staining of a canine osteochondral allograft in culture for 56 days. Calcein AM is green and metabolized by viable cells. The image demonstrates excellent viability of the chondrocytes in the articular cartilage.

Grafts were matched to recipients based on the relative size of the donor and recipient joints using measurements made on calibrated radiographs. Typically size was measured as the width of the articular surface (e.g., femoral condyles) in the frontal plane; size discrepancies were limited to <3 mm, meaning that the size of the donor joint could only differ from the measured size of the recipient joint by a 2.9 mm or less. Osteochondral tissues from the same donor, but which were not going to be transplanted but rather assessed for chondrocyte viability, were preserved in parallel to the tissues intended for transplantation. The tissues that were preserved for assessing chondrocyte viability were assayed for viable cells with Calcein AM and dead cells with the SYTOX™ blue assay (both from Thermo Fisher Scientific, Waltham, MA) to ensure viable chondrocyte density was >70% of established reference range at time of clearance (Figure 1) (15, 16). Handling of OCA was done in compliance with US Food and Drug Administration guidelines for human OCAs (section 361 of the Public Health Services Act). All OCAs were implanted within 56 days of donor death because a previous study has shown that all MOPS®-preserved canine donor tissue exceeds minimum essential viable chondrocyte density up to 56 days following tissue recovery (30).

**Figure 2:**
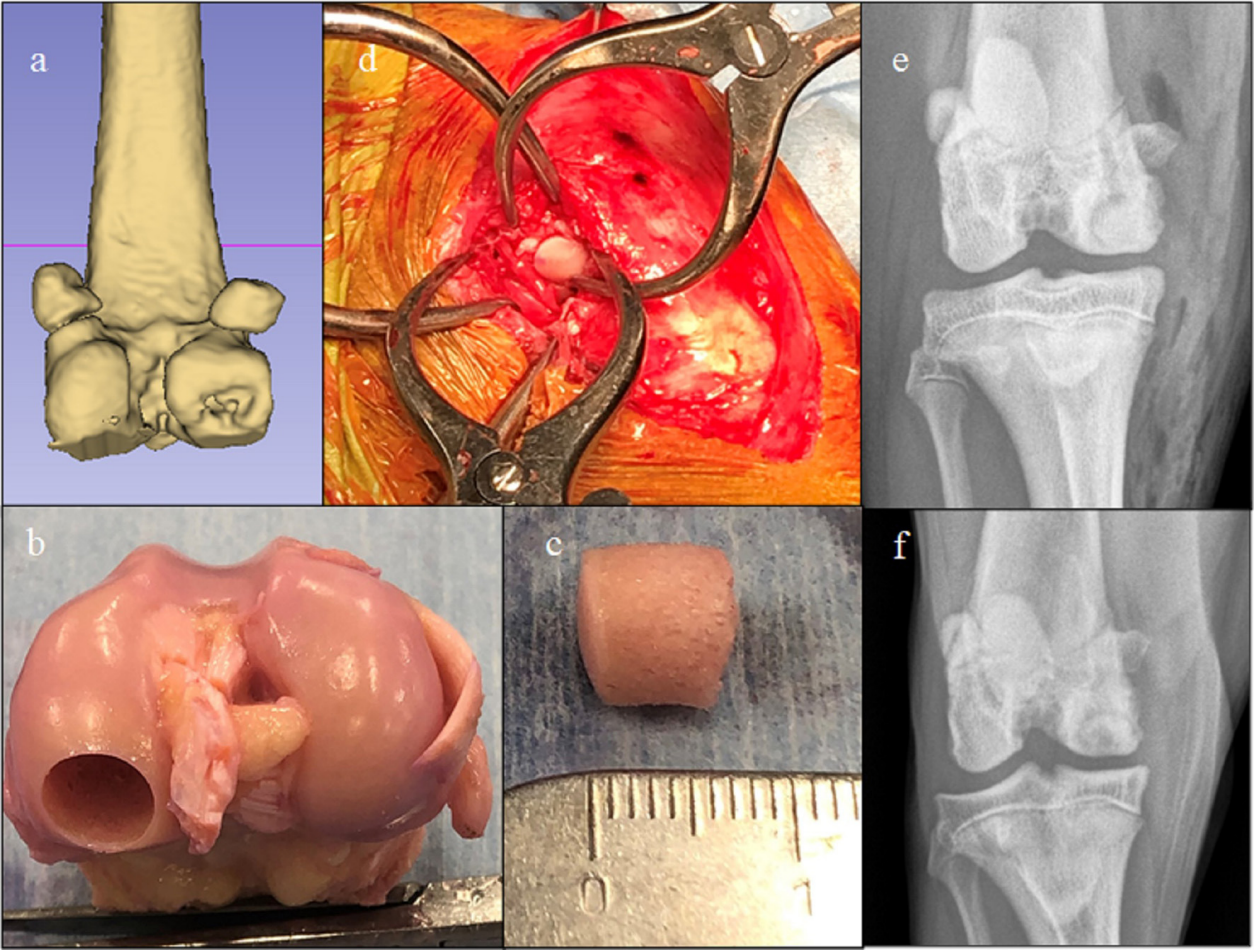
Images of a dog with a medial femoral osteochondritis dissecans lesion treated with an osteochondral allograft (OCA). **(a)** Pre-operative CT 3-D reconstruction showing the defect. **(b)** Donor stifle, **(c)** Harvested OCA plug, **(d)** Intra-operative image showing graft implantation through a caudomedial arthrotomy, **(e)** Immediate post-operative radiograph, and **(f)** 8-week post-op-radiograph. This dog developed a seroma that resolved without treatment and then achieved a full functional outcome.

**Figure 3:**
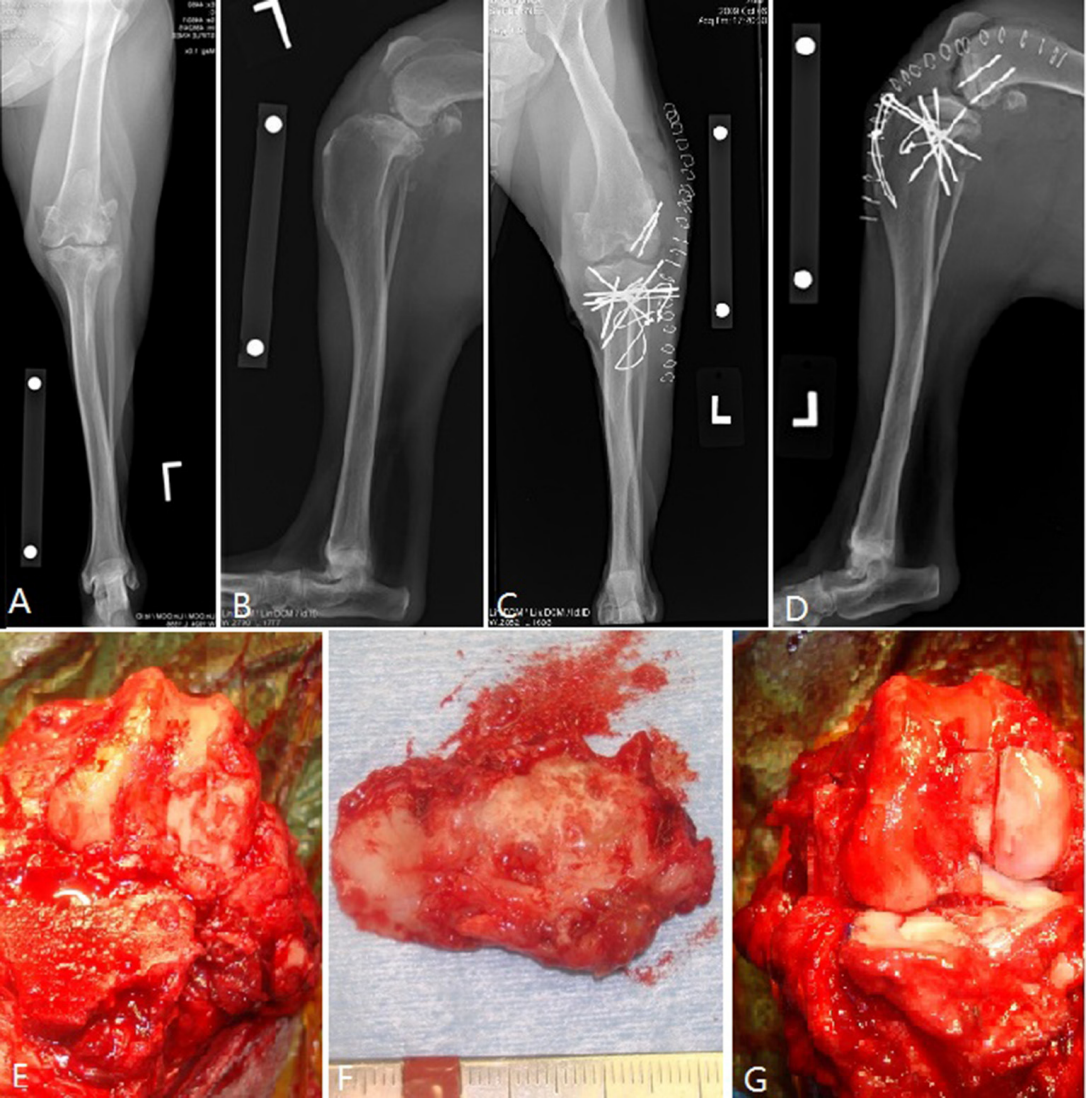
Osteochondral allograft resurfacing of the canine tibial plateau and lateral femoral condyle with transplantation of fresh meniscal allografts in a dog with numerous previous surgeries for cranial cruciate ligament and meniscal deficiency. **(A,B)** Pre-operative radiographs; **(C,D)** post-operative radiographs. Note a decrease in tibial plateau angle performed to address the cranial cruciate ligament deficiency. **(E–G)** Intra-operative images showing removal of the diseased tibial plateau and what remained of the menisci and the post-transplantation images showing the OCA tibial plateau and lateral femoral condyle *in situ* along with the two allograft menisci. This dog had seroma formation and had implant migration which required implant removal after integration of the grafts. The dog achieved a full functional outcome.

**Figure 4:**
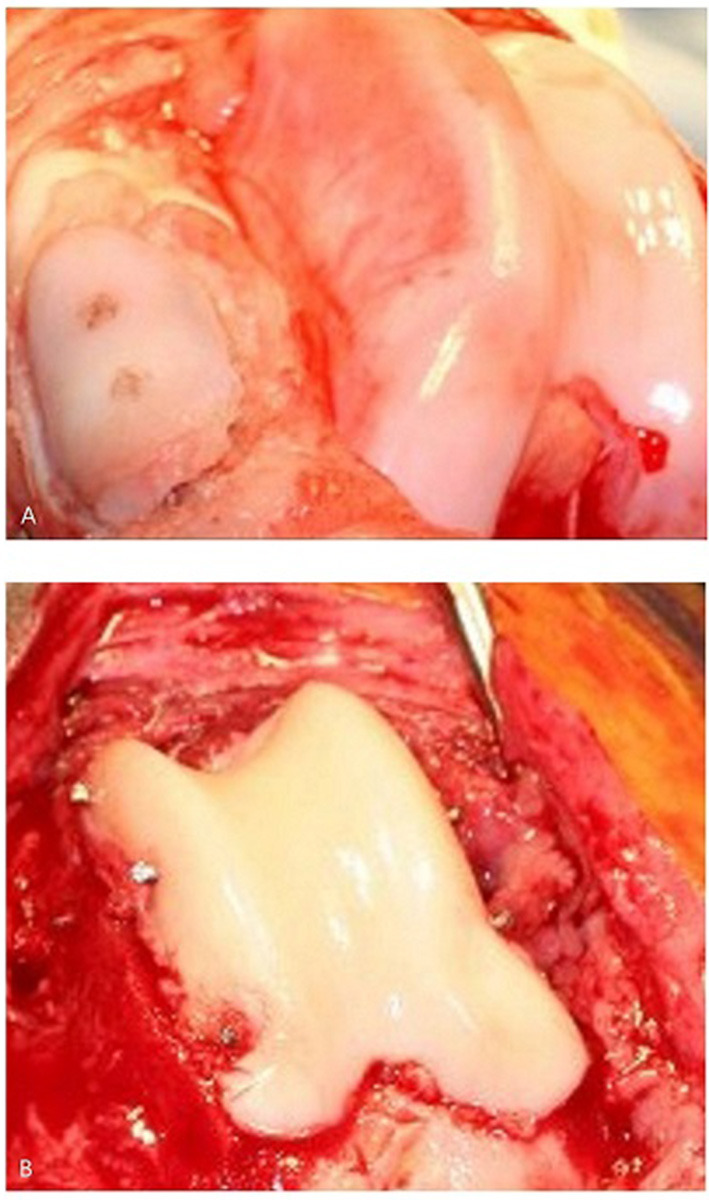
**(A)** Canine patella resurfacing (image is showing patella resurfacing technique in a cadaver) and **(B)** trochlear re-surfacing of a canine patient. The patient in **(B)** achieved an acceptable functional outcome.

**Figure 5:**
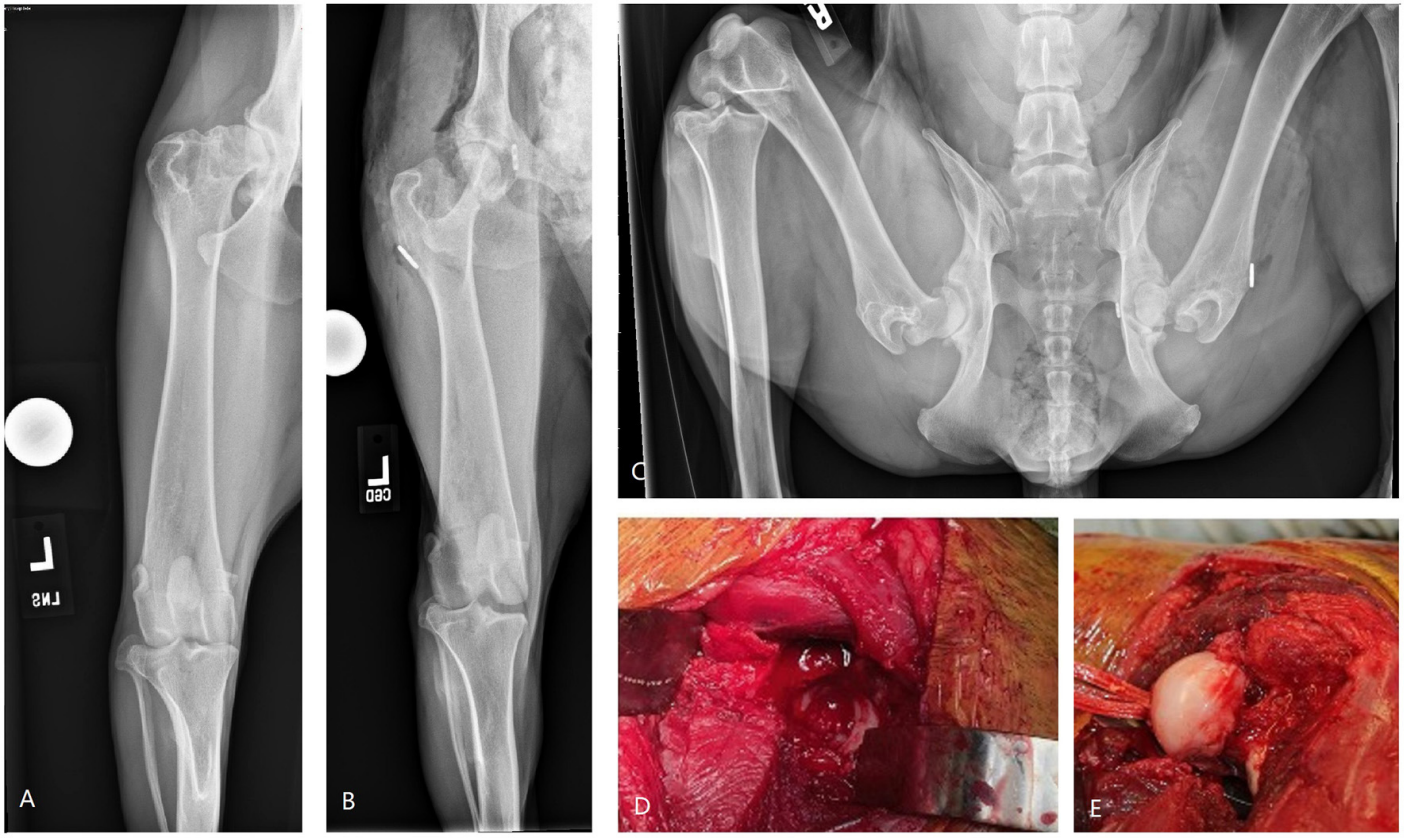
Images of bipolar osteochondral allograft transplantation to treat end-stage coxofemoral osteoarthritis. **(A)** Pre-operative radiograph, **(B,C)** immediate post-operative radiographs, **(D)** acetabular OCA placement; **(E)** femoral head OCA placement with toggle pin construct to maintain femoral head reduction. This dog suffered femoral neck fracture and was revised with a femoral head and neck excision and was classified as having a major complication and unacceptable outcome.

OCA transplantation techniques were dependent upon indication and surgeon preference. One ulnar lesion and 19 femoral condylar osteochondritis dissecans (OCD) lesions were treated with OCA plugs (6–10-mm diameter, ~5–8 mm depth) using press-fit technique (Figure 2) and either Osteochondral Autograft Transplant Systems instrumentation (Arthrex Vet Systems, Naples, FL) or COR^®^ Precision Targeting System instrumentation (COR^®^ Mitek Sports Medicine, DePuy Synthes Vet, West Chester, PA). One humeral head OCD lesion was treated with a 25 mm diameter OCA plug (~7 mm depth) using press-fit technique and ACT System instrumentation (ACT™; Musculoskeletal Transplant Foundation, Edison, NJ). All other defects were treated using custom-cut patient-specific shell (5–7 mm thick) OCAs stabilized with 0.045", 0.062" Kirschner wires, 2.0 mm cortical screws, bioabsorbable pins (Arthrex, Inc., Naples, FL, USA), or bioabsorbable nails (ConMed Linvatec, Utica, NY, USA) (Figures 3–5). Fresh meniscal allograft transplants, if performed in conjunction with femoral condyle or tibial plateau resurfacing, were performed either using a bone plug technique with suspensory fixation or included as part of the tibial OCA transplant (34). Immediately prior to implantation, OCA bone was irrigated with isotonic saline to dilute marrow elements. Post-operative management protocols included oral analgesia, typically involving oral non-steroidal anti-inflammatory medications with or without additional analgesics such as Gabapentin, for 2–4 weeks and leash-only exercise for 8 weeks following surgery.

Signalment, treated joint, underlying pathologic change, type of graft used, size and number of grafts, surgical approach, and methods of graft fixation were extracted from the medical record. Participating surgeons, with client input, classified complications and outcomes for the patients they had treated as follows. Complications that did not necessitate any treatment were classified as “trivial.” An example of such type of complication was seroma formation that resolved without treatment. Complications that completely resolved with non-surgical management were classified as a “non-surgical complication.” An example of such type of complication was suspicion of a superficial incisional infection that resolved with a 10-day course of oral antibiotics. Complications that warranted a minor surgical procedure, but that did not require revision surgery and did not preclude obtaining acceptable function of the graft, were categorized as “minor surgical.” An example of a minor surgical complication would be removal of Kirschner wires a graft became integrated and stable. Finally, failure of a graft that warranted surgical revision was considered a “major” complication.

Outcomes were classified as full, acceptable, or unacceptable function (35). Full function included returning to the full intended level and duration of activities and performance from pre-injury or pre-disease status without use of medication. Acceptable function was defined as return to intended activities and performance from pre-injury or pre-disease status that is limited in level or duration and/or requires medication to achieve. Unacceptable function was classified as all other outcomes.

Descriptive statistics were calculated to report means, standard deviations, ranges, and percentages. Success and complication rates were compared between medial and lateral femoral condyle OCD lesions, between dogs that had their stifle vs. another joint treated, between dogs that had only one surface (i.e., unipolar) vs. two articular surfaces treated (i.e., bipolar), and between dogs that had only plugs used in their treatment vs. those that had patient-specific custom shell grafts used. All comparisons were performed using a Fishers Exact-Test. Significance was set at *P* < 0.05.

## Results

Thirty-five joints in 33 dogs were treated by eight veterinarians at nine centers across the US and Canada; two dogs were treated bilaterally (Table 1). There were five Labrador retrievers, four each of German Shepherd dogs and mixed breeds, three Rottweilers, two each of Golden Retrievers, Border Collies, and Pointer, and one each of Akita, Australian Shepherd, Cane Corso, Chesapeake Bay Retriever, Great Dane, Leonberger, Mastiff, Miniature Schnauzer, Ovcharka, Samoyed, and Viszla. There were 13 intact males, 10 castrated males, three intact females, and seven spayed females. Mean age at time of OCA transplantation was 32.5 months (SD 32 months, range 7 mos−13 years). Mean follow-up duration was 28 months (SD 23 months, range 6 weeks−8 years).

**Table 1:** List of all cases treated with osteochondral allograft transfer.

**Breed**	**Age (mos)**	**Follow-up (mos)**	**Indication**	**Grafts**	**Size**	**Techn**.	**Complications**	**Outcome**	**Surgical revision**
**Medial femoral condyle OCD cases**									
Bord Collie	18	12	MFC OCD	1 Plug	10 mm	OATS	None	Full	None
Aus Shepherd	17	12	MFC OCD	1 Plug	10 mm	OATS	None	Full	None
Mix	7	6	MFC OCD	1 Plug	8 mm	OATS	Seroma^a^	Full	None
Labrador	14	68	MFC OCD	2 Plugs	10, 8 mm	OATS	Seroma^a^	Full	None
G Dane	16	22	MFC OCD	2 Plugs	10, 8 mm	OATS	Seroma^a^	Full	None
Labrador	14	31	MFC OCD	1 Plug	10 mm	OATS	Seroma^a^	Full	None
Viszla	11	40	MFC OCD	1 Plug	8 mm	OATS	Seroma^a^	Full	None
Mastiff	35	24	MFC OCD	2 Plugs	10, 8 mm	OATS	None	Full	None
Rottweiler	20	15	MFC OCD	1 Plug	10 mm	OATS	Superficial infection^b^	Acc.	None
GSD	26	24	MFC OCD	1 Plug	10 mm	OATS	None	Acc.	None
**Lateral femoral condyle OCD cases**									
Bord Collie	11	29	LFC OCD	1 Plug	8 mm	OATS	None	Full	None
GSD	15	13	LFC OCD	1 Plug	10 mm	OATS	Radiographic progression of OA, untreated^a^	Acc.	None
Lab	15	28	LFC OCD	1 Plug	10 mm	OATS	None	Acc.	None
Golden Ret	23	26	LFC OCD	2 Plugs	8 mm × 2	OATS	None	Full	None
Labrador	18	27	LFC OCD	1 Plug	10 mm × 8 deep	OATS	None	Acc.	None
Cane Corso	29	5	LFC OCD, bilateral (L)	1 Plug each	10 mm	OATS	None	Acc.	None
Cane Corso	29	5	LFC OCD, bilateral (R)	1 Plug each	10 mm	OATS	None	Acc.	None
GSD	38	13	LFC OCD, bilateral (L)	1 Plug	10 mm	OATS	Cartilage delamination^d^	Unacc.	Debridement, OA management
GSD	38	9	LFC OCD, bilateral (R)	1 Plug	10 mm	OATS	Cartilage delamination^d^	Unacc.	Debridement, OA management
**Complex articular reconstructions**									
Golden Ret	19	19	LFC OCD	Anatomic	Full LFC	Custom cut	None	Full	None
Leonberger	15	2	LFC OCD	Anatomic	Partial condyle	Custom cut	Non-union^d^	Unacc.	Debridement, OA management
Ovcharka	38	49	Humeral head OCD	1 Plug	25 mm	MTF OCA	None	Acc.	None
Samoyed	17	43	MHC Dysplasia	Anatomic	Full MHC	Custom cut	None	Full	None
Rottweiler	26	17	Talar OCD	Anatomic	Talar defect area	Custom cut	None	Acc.	None
Pointer	20	41	Patellar fracture	Anatomic	Full patella	Custom cut	None	Full	None
Min Schnauz	161	9	Proximal radial OSA	Anatomic	Full radial head	Custom cut	None	Acc.	None
Mix	36	7	Shearing lesion chronic	Anatomic	Full patella (with patellar tendon and TT)	Custom cut	None	Acc.	None
GSD	48	1.5	CHD	Bipolar anatomic	Full femoral head, acetabulum	Custom cut	Femoral neck fracture^d^	Unacc.	FHNE
Pointer	85	99	Knee PTOA, meniscetomies	Bipolar anatomic	Full plateau, menisci, MFC	Custom cut	Implant migration^c^	Full	Pin removal
Labrador	131	30	Elbow dysplasia PTOA	Bipolar anatomic	full MHC, MCP 6 mm plug	Custom cut, OATS	Delayed union^a^	Acc.	None
Akita	20	60	Trochlear dysplasia, MPL, PTOA	Bipolar anatomic	Full patella, trochlea	Custom cut	None	Acc.	None
Chesapeake	47	83	Knee PTOA, meniscectomy	Bipolar anatomic	Full MFC, medial meniscus	Custom cut	None	Full	None
Rottweiler	25	55	Talar OCD	Bipolar anatomic	Full talus, distal tibia	Custom cut	Implant irritation^a^	Acc.	None
Mix	22	34	MFC dysplasia, meniscal deficiency	Bipolar anatomic	Full MFC, medial meniscus	Custom cut	Implant migration^c^	Acc.	Pin removal
Mix	23	1.5	Shoulder dysplasia	Bipolar anatomic	Full humeral head, glenoid	Custom cut	Non-union^d^	Unacc.	Shoulder arthrodesis

*Techn., technique; OA, osteoarthritis; PTOA, post-traumatic OA; OCD, osteochondritis dissecans; MTF, Musculoskeletal Transplant Foundation; MHC, medial humeral condyle; MCP, medial coronoid process; MFC, medial femoral condyle; TT, tibial tuberosity; OATS, osteochondral autograft transfer system; FHNE, femoral head and neck excision; Anatomic, patient-specific custom-cut shell graft rather than an osteochondral plug; Acc, acceptable; Unacc, unacceptable.*

*Superscripts denote complication classification.*

a *Trivial.*

b *Non-surgical.*

c *Minor surgical.*

d *Major*.

Ten medial femoral condylar (MFC) OCD lesions in 10 dogs were treated with plugs (Figure 2). All 10 lesions were treated using OATs instrumentation. Five were treated with one 10 mm plug, two were treated with one 8 mm plug, and three were treated with both a 10 and 8 mm plug. Mean follow-up time was 25.4 months (SD 18; range 6–68 months). There were five dogs that developed seromas only and which resolved without treatment; these were classified as trivial complications. One dog had a non-surgical complication, a presumed superficial incision infection, that resolved with a short course of oral antibiotics. There were no minor surgical or major complications. Outcomes were classified as full function in eight cases and acceptable in two cases with no unacceptable outcomes for a success rate of 10/10.

Nine lateral femoral condyle (LFC) OCD lesions in seven dogs were treated using plugs. Seven stifles were treated using one 10 mm plug, one stifle was treated with one 8 mm plug, and one stifle was treated with two 8 mm plugs. Mean follow-up time was 16.8 months (SD 10.4; range 5–29 months). One dog had a trivial complication that didn't necessitate treatment. Two stifles in one dog had delamination and/or necrosis of the cartilage layer in both stifles that was confirmed with second-look arthroscopy; classified as “major” complications. This dog was managed non-operatively for OA and both stifles were classified as having an unacceptable outcome. In total there were 2/9 joints that had full function, 5/9 with acceptable function, and 2/9 that had unacceptable function for a success rate of 7/9.

Sixteen applications of OCA transplantation were deemed “complex” articular reconstructions. One dog had a 25 mm diameter osteochondral plug transplanted for treatment of a humeral head OCD. Fifteen cases had anatomic partial or total joint reconstructions performed with custom cuts of recipient bone and OCA (Table 1; Figures 3–5). Median follow-up time was 34 months (SD 30; range 6 weeks−99 months) with short follow-up times (6 weeks) for dogs with short-term failures of the OCA transplantation. There were 2 trivial complications that included delayed integration of the osseous portion of the graft based on radiographs (i.e., persistent radiolucent boundary between the donor cancellous bone and the surrounding native bone) and another case with presumed implant irritation. Both resolved without treatment and both dogs had acceptable outcomes. There were 2 minor surgical complications which involved implant removal after OCA integration was complete. The grafts survived in both dogs and they had full and acceptable function, respectively. There were 3 major complications with associated unacceptable function.

The 3 major complications included a German Shepherd dog with OCA transplantation of the femoral head and acetabulum to treat severe coxofemoral osteoarthritis (Figure 5). A toggle pin procedure was simultaneously performed to maintain reduction of the femoral head. That dog suffered a femoral neck fracture and so a femoral head ostectomy (FHO) was performed. Function of the OCA transplantation was classified as unacceptable.

Another dog had humeral head and glenoid OCA transplants for a bipolar reconstruction and there was failure of graft integration on the glenoid side. The dog had a shoulder arthrodesis performed. This was considered a major complication and the outcome of the OCA transplantation was classified as unacceptable.

A third dog had a partial LFC replaced with a custom-cut allograft. The graft failed to integrate and became loose. A second surgery was performed to debride the graft and the dog was treated non-operatively thereafter. This was classified as a major complication and the outcome of the OCA transplantation was classified as unacceptable.

In total, for these 16 complex reconstructions, there were five cases with full function, 8 acceptable outcomes, and 3 unacceptable outcomes for a success rate of 13/16. Six of 8 cases with bipolar reconstructions were successful and 7 of 8 cases with unipolar reconstructions were successful.

There were no statistically significant differences in success and complication rates between medial and lateral femoral condyle OCD lesions, between stifle joints and other treated joints, between unipolar and bipolar treatment, or between use of plugs only vs. custom-cut shell grafts. The lowest *p*-value obtained was *p* = 0.06 and was the comparison in complication rates between unipolar vs. bi-polar reconstruction with bipolar reconstructions having a numerically higher proportion of cases with complications. These data are shown in Table 2.

**Table 2:** Comparison of success and major complication rates for treatment cohorts undergoing OCA transplantation.

**Comparison**	**Success rate**	***p*** **-value**	**Major complication rate**	***p*** **-value**
MFC OCD (*n* = 10)	10/10 (100%)	0.21	1/10 (10%)	0.59
LFC OCD (*n* = 9)	7/9 (78%)		2/9 (22%)	
Stifle (*n* = 27)	24/27 (89%)	0.57	6/27 (22%)	1
All other joints (*n* = 8)	6/8 (75%)		2/8 (25%)	
Unipolar (*n* = 27)	24/27 (89%)	0.57	4/27 (15%)	0.06
Bipolar (*n* = 8)	6/8 (75%)		4/8 (50%)	
Plug (*n* = 20)	18/20 (90%)	0.63	3/20 (15%)	0.25
Custom cut anatomic (*n* = 15)	12/15 (80%)		5/15 (33%)	

*OCD, Osteochondritis dissecans*.

## Discussion

The purpose of this study was to report data on complications and outcomes associated with OCA transplantation for treatment of chondral and osteochondral lesions in dogs. Since this was a retrospective study without other treatment groups, it is impossible to draw conclusions regarding inferiority or superiority of OCA transplantation in comparison to current treatments. However, with a relatively high success rate, we believe that comparison of OCA transplantation to other treatments, such as non-surgical management, osteochondral autografting, or synthetic resurfacing with focal, unicompartmental, or total joint replacement systems, is warranted.

A previous study reported outcomes with OAT for treating MFC OCD in six stifles (5 dogs). In that study all owners were “very satisfied,” while one dog had persistent pain and lameness, consistent with a success rate of 5/6 (36). Two complications were reported (2/6). The data from our study show a numerically higher success rate with OCA transplantation. As for LFC OCD lesions, a clinical study assessed OAT in 12 stifles in 10 dogs and found that 4 joints had complications (4/12). Two of the 10 dogs regained full function and the remaining 8 dogs had acceptable function (9). That success rate (12/12) is numerically greater than that obtained in this study of OCA transplantation (7/9). However, these data are inadequate to demonstrate inferiority of OCA transplantation vs. OATs for treatment of MFC or LFC OCD. We conclude that both OAT and OCA transplantation are feasible treatment options for focal OCD lesions of either femoral condyle in dogs.

Another surgical option for treatment of focal cartilage defects in dogs involves implantation of synthetic “plugs” (SynACART, Arthrex Vet Systems, Naples, FL). The synthetic implant is appealing because of “off-the-shelf” availability, technical simplicity, and lack of donor site morbidity. In the clinical canine study assessing use of the commercially available second-generation implant for treatment of stifle OCD, there were eight lesions treated in five dogs, with one MFC lesion and seven LFC lesions. Seven out of eight stifles had a successful outcome without complication while one LFC implant required removal due to persistent infection. This equates to a success rate of one of one for MFC lesions and 6/7 LFC cases (13). Definitive conclusions regarding superiority or inferiority cannot be drawn between OCA transplantation and this implant based upon these data.

As for the cases that had “complex” reconstructions using custom-cut patient-specific shell grafts or a large (25 mm) plug, OCA transplantation allowed for successful treatment of a relatively high proportion (13/16) of these patients. We conclude that OCA transplantation can be a viable treatment option worth considering. Furthermore, there are some appealing characteristics of OCA transplantation including that it can be performed with common instrumentation, it enables treatment of some joints for which commercially available joint replacements are not available, OCAs are available from at least two tissue banks in the United States, and OCAs cost less (cost are typically $500–1,000 per joint) than total joint replacement prostheses. However, there are numerous potential disadvantages of OCA transplantation for complex articular reconstructions. Some of the procedures performed in this study were technically demanding. It is important to underscore that the relatively high success rate with the complex reconstructions in this study may not be representative of what most veterinary surgeons would obtain. We cannot provide more specific guidelines as to which cases are more, or less likely, to be treated with OCA transplantation because there were no statistically significant comparisons in success or complication rates between medial vs. lateral femoral OCD lesions, stifle vs. other joint, plug vs. custom-cut grafts, or between unipolar vs. bi-polar reconstructions. However, it is quite feasible that the lack of statistically significant differences in some of these comparisons could have been attributable to a type II statistical error. The comparison between unipolar and bipolar treatments reached a *p*-value of 0.06 with bi-polar reconstructions having complications in a higher proportion of dogs. It is plausible that bipolar reconstructions are more challenging and have a higher complication rate. Further research will need to be done to identify factors that influence the success and complication rates of OCA transplantation in dogs including what joint and type of pathology are being treated, the severity of the cartilage damage and duration of clinical signs prior to treatment, and the type of grafts used and surgical technique, among other variables.

Additional concerns with OCA transplantation include the potential for immune responses, disease transmission, and longevity of the reconstructions. Immune-rejection has not been appreciated in human patients or research dogs, and did not occur in any of the client-owned dogs in this study (18, 19, 29, 37). Similarly, infectious disease transmission was not recognized in any of the dogs of this study and seems unlikely with appropriate donor and tissue screening. Finally, while mean follow-up time for dogs in this study was over 2 years, the duration of functional outcomes after OCA transplantation in dogs cannot be established from these data. This is a relevant question as a significant proportion of dogs with chondral and osteochondral lesions requiring surgical treatment are young and may have 10+ years of life remaining. Osteochondral allograft transplantation in people is associated with functional survival rates of up to 93% at 10 years and up to 84% at 15 years, which bodes well for canine patients (28, 38–40). However, long term survivorship data for canine patients must be collected before any related conclusions can be made.

These are the first data on complications and outcomes following OCA transplantation to treat naturally occurring disease in client-owned dogs. As this was a retrospective study, there were several limitations including lack of objective outcomes measures, dependency on medical records for quantifying complications and outcomes, a heterogeneous patient population, and lack of comparison groups treated by alternative methods. In addition, second-look arthroscopy was not performed on patients that had acceptable or full function as second-look arthroscopy in these patients can be financially and ethically challenging. Consequently, the true outcome and viability of the transplanted cartilage in these patients remains undocumented. However, while acknowledging this limitation, readers should also understand that follow-up arthroscopy is not available on all patients in studies on OATs or synthetic implants (SynACART, Arthrex Vet Systems, Naples, FL) either. In any event, these data do not enable us to draw conclusions as to whether OCA transplantation is superior or inferior to alternative treatment options. However, we do conclude that results with OCA transplantation from this study justify further evaluation of OCA application in dogs and that OCA transplantation may have a place in canine medicine as it does in treatment of chondral and osteochondral defects in humans.

## Data Availability Statement

The original contributions presented in the study are included in the article/supplementary material, further inquiries can be directed to the corresponding author.

## Ethics Statement

The animal study was reviewed and approved by University of Missouri Animal Care and Use Committee. Written informed consent was obtained from the owners for the participation of their animals in this study.

## Author Contributions

AS: responsible for all graft harvest, preservation, evaluation, and manuscript review. SM, MPK, MG, SK, MK, AC, and JC: manuscript review. SF: writing the manuscript. All authors contributed to the article and approved the submitted version.

## Conflict of Interest

JC is the director of the Thompson Laboratory for Regenerative Orthopedics. JC and AS are patent holders for the Missouri Osteochondral Preservation System (MOPS^®^) and receive royalties associated with use of MOPS^®^ in humans. The remaining authors declare that the research was conducted in the absence of any commercial or financial relationships that could be construed as a potential conflict of interest.

## Publisher's Note

All claims expressed in this article are solely those of the authors and do not necessarily represent those of their affiliated organizations, or those of the publisher, the editors and the reviewers. Any product that may be evaluated in this article, or claim that may be made by its manufacturer, is not guaranteed or endorsed by the publisher.
