# Corrigendum: Outcomes Associated With Osteochondral Allograft Transplantation in Dogs

**DOI:** 10.3389/fvets.2021.843760

**Published:** 2022-01-21

**Authors:** Samuel P. Franklin, Aaron M. Stoker, Sean M. Murphy, Michael P. Kowaleski, Mitchell Gillick, Stanley E. Kim, Michael Karlin, Alan Cross, James L. Cook

**Affiliations:** ^1^Colorado Canine Orthopedics and Rehab, Colorado Springs, CO, United States; ^2^Thompson Laboratory for Regenerative Orthopaedics, University of Missouri, Columbia, MO, United States; ^3^WestVet Animal Emergency and Specialty Center, Garden City, ID, United States; ^4^Department of Clinical Sciences, Cummings School of Veterinary Medicine, Tufts University, North Grafton, MA, United States; ^5^Toronto Veterinary Emergency and Referral Hospital, Toronto, ON, Canada; ^6^Department of Small Animal Clinical Sciences, University of Florida, Gainesville, FL, United States; ^7^BluePearl Pet Hospital, Atlanta, GA, United States

**Keywords:** osteochondral, allograft, transplants, cartilage repair, dogs, osteochondrosis, osteochondritis dissecans

In the original article, the letters “XXX” were published, where specific information should be substituted, in the **Materials and Methods** section page 1, paragraph 1, page 1, paragraph 2 and page 3, paragraph 3. The corrected paragraphs appear below

**MATERIALS AND METHODS, paragraph 1**

This retrospective study involved acquisition of data for all dogs undergoing OCA transplantation using OCAs provided by a single canine tissue bank (The Thompson Laboratory for Regenerative Orthopaedics, University of Missouri) and for whom follow-up data were available.

**MATERIALS AND METHODS, paragraph 2**

Osteochondral tissues were recovered using strict aseptic technique immediately following euthanasia (i.e., within 30 min of euthanasia), measured, and immediately placed in a proprietary medium and stored in accordance with a validated protocol (Missouri Osteochondral Preservation System; MOPS®) (15, 16, 31).

**MATERIALS AND METHODS, paragraph 3**

All OCAs were implanted within 56 days of donor death because a previous study has shown that all MOPS®-preserved canine donor tissue exceeds minimum essential viable chondrocyte density up to 56 days following tissue recovery (30).

The authors apologize for these errors and state that these do not change the scientific conclusions of the article in any way. The original article has been updated.

## Publisher's Note

All claims expressed in this article are solely those of the authors and do not necessarily represent those of their affiliated organizations, or those of the publisher, the editors and the reviewers. Any product that may be evaluated in this article, or claim that may be made by its manufacturer, is not guaranteed or endorsed by the publisher.

